# Who are the ostomy patients and caregivers attending Portuguese community pharmacies? A cross-sectional study

**DOI:** 10.1186/s12913-020-05765-7

**Published:** 2020-10-02

**Authors:** Mariana Romão, Débora Figueira, Heloísa Galante, José Guerreiro, Sónia Romano

**Affiliations:** Centre for Health Evaluation & Research/Infosaude – National Association of Pharmacies (CEFAR/IS-ANF), Rua Marechal Saldanha 1, 1249-069 Lisbon, Portugal

**Keywords:** Ostomy, Community pharmacies, Caregivers, Health Services accessibility, Satisfaction

## Abstract

**Background:**

In 2017, ostomy patients gained access to ostomy products in community pharmacies that are fully reimbursed by the Portuguese National Health Service. This impacted the daily lives of people with ostomy and opened a new market of products and services for pharmacies. However, little is known about the sociodemographic and clinical profile of ostomy patients. This study aims to characterize people with ostomy and their caregivers, evaluate access and satisfaction with the pharmacy and explore participants’ expectations regarding services and counselling.

**Methods:**

This was an observational, cross-sectional, multicentre study involving pharmacy users who acquired ostomy products in Portuguese community pharmacies. Data were collected through a confidential self-report questionnaire between June and August 2019.

**Results:**

Approximately 56% of the participants were ostomy patients, of whom 65.9% were men. The average age of participating ostomy patients was 65.5 years old (SD = 12.9), and near 80% were retired/pensioners. Caregivers were mostly women (81.7%). More than half of the caregivers were employed and acquired products for a direct family member. Three in every four surgical interventions were consequences of cancer. Intestinal ostomy was the most common intervention (78.3%). More than 93% were satisfied with the acquisition of ostomy products at the pharmacy. Approximately 48.2% of ostomy patients received care from a specialized nurse.

**Conclusion:**

This study describes the profile of people with ostomy and their caregivers who attend community pharmacies in Portugal. Participants’ perceptions of the utility of different proposed services and pharmacist knowledge, as well as the low coverage of ostomy nursing care, highlight the opportunity for an extended role of pharmacists among this group.

## Background

A stoma is an artificial opening between a cavity or canal and the surface of the body [1] that creates a bypass to facilitate impaired vital function, such as the elimination of excretions (e.g., colostomy, ileostomy, urostomy), breathing (e.g., tracheostomy) or feeding (e.g., gastrostomy, jejunostomy) [2, 3]. After the formation of an elimination stoma, ostomy patients must carry a pouching system, which is composed of a skin barrier and a pouch where bodily waste is collected. The main goal of the pouching system is to allow ostomy patients to perform common daily activities [4]. Handling this condition can be quite complicated, and caregivers play an essential role in the daily lives of people for whom the condition poses more challenges.

Data on ostomy patients in Portugal are scarce. It is estimated that there are approximately 20,000 ostomy patients in Portugal [5]. Information on their sociodemographic and clinical characteristics on a nationwide scale is limited as well.

Access to ostomy products has changed in the last couple of years. Before 2017, there was no standardized procedure to access these products, and patients had to navigate a complex system involving primary care facilities, suppliers, pharmacies and patients’ organizations, among others [6]. Ostomy products were 90% reimbursed [7], but patients often had to wait several months for their out-of-pocket expenses to be refunded [6]. This system created inequalities in access, reimbursement and social integration [6, 8]. In 2017, the government enacted a law – effective since April 2017 – to improve the control of and equitable access to these products. The new model established clear rules and procedures for the prescription, dispensing and reimbursement of ostomy products. Ostomy patients who present a prescription at a community pharmacy now have access to their medical devices without out-of-pocket payments, and the costs are fully covered by the Portuguese National Health Service (NHS) [9, 10].

This paradigm placed community pharmacies in a prominent position, paving the way for a market that demands a whole new skillset and the development of tailored pharmacy services. In the same year, a study was conducted to assess the impact of this new legislation. The study examined multiple dimensions, such as access to pharmacy and ostomy products, impacts on expenditure, and participants’ satisfaction with and perceptions of pharmacists. The results suggested that pharmacies should adopt a strategy to establish a closer relationship with ostomy patients [11].

At the time of the present study, 2 years have elapsed since ostomy products started being dispensed with fully covered expenses in Portuguese community pharmacies. The new paradigm is likely to have reshaped ostomy patients’ and caregivers’ relationships with pharmacies. This study aims to characterize ostomy patients and their caregivers, as well as evaluate access to ostomy products and ostomy patients’ and caregivers’ satisfaction with pharmacies. Another objective is to explore ostomy patients’ and caregivers’ unmet needs from and expectations of pharmacies regarding counselling and other provided services.

## Methods

### Study design

This was an observational, cross-sectional, multicentric study of individuals who acquired ostomy products (ostomy patients or caregivers). The study took place in community pharmacies affiliated with the National Association of Pharmacies (ANF) located in continental Portugal and the Autonomous Region of Madeira. Only pharmacies that registered ostomy product sales in 2018 were invited to participate in the study (*n* = 2314).

Participants were enrolled between June 22 and August 11 of 2019. Customers who usually went to the pharmacy to obtain ostomy products and were 18 years or older, literate, and able to sign an informed consent form were eligible to participate in the study.

### Sample size

On average, 6 people per month acquire ostomy products at community pharmacies. Assuming the participation of approximately 200 pharmacies and a refusal rate of 50% of the patients, 1 month was expected to be enough to recruit the estimated sample size of 375 patients. This would ensure a maximum absolute error of 5% for the estimated sample with a 95% confidence level.

### Data sources

Participating pharmacies received a pop-up alert on Sifarma® dispensing software when delivering an ostomy product. This alert reminded the pharmacist to check the inclusion criteria and invite customers to participate in the study; this strategy was also intended to decrease participant self-selection. Those who agreed to participate were required to sign an informed consent form. If a client did not agree to participate, pharmacists were asked to complete a refusal form with the customer’s sociodemographic information and reason for refusal.

Data were collected through a semi-structured questionnaire specifically developed for the study, comprised of 28 questions regarding sociodemographic information, clinical history, access to pharmacy and ostomy products, and satisfaction with and expectations of the pharmacy and suggested services (see Additional file 1). The questionnaire could be completed by either ostomy patients or their caregivers. When caregivers completed the questionnaire, they were asked to provide their own sociodemographic characteristics as well as the characteristics of the ostomy patient under their care (age, gender, employment status and degree of kinship with the ostomy patient). To ensure data confidentiality, participants received an envelope in which to place the questionnaire before returning it to the pharmacist.

### Statistical analysis

Customers who declined to participate were characterized and compared with the respondents to check for differences and sampling bias. The distribution of participating pharmacies was compared with the population of pharmacies to check for differences in the administrative health regions and rural and urban areas.

A descriptive analysis of the collected variables was performed, and the results were stratified by customer type (ostomy patient or caregiver).

Ostomy patients who went to the pharmacy on their own were classified as “autonomous”, and those represented by a caregiver were classified as “dependent”. Differences in sociodemographic characteristics between these groups were checked. Age at the time of the surgery was calculated for each ostomy patient based on his or her current age and the amount of time since the surgery.

Categorical variables were summarized by absolute and relative frequencies. Percentages were calculated using the total number of respondents to each question as the denominator (missing values were excluded from the analysis). Continuous variables were summarized using measures of central tendency and dispersion, such as the mean, median and standard deviation. Subpopulations were compared using the chi-square test or Fisher’s exact test for categorical variables and the t-test or ANOVA for continuous variables, with a significance level of 5%. Data analysis was performed using MS Office and SAS Software (SAS Institute, Cary, NC, USA). The STROBE checklist was used to ensure appropriate and transparent reporting of the study results [12].

## Results

Of the 2314 pharmacies invited to participate in this study, 185 pharmacies (8.0%) willingly accepted and recruited a total of 412 people. The participating pharmacies were representative of the Portuguese population of community pharmacies. The analysis showed no significant differences (*p* > 0.05) in the distribution of participating pharmacies compared to that of the pool of invited pharmacies in terms of either administrative health regions or rural and urban areas. A total of 48 people refused to participate in the study. The main reason for refusal was lack of time to participate (58.3%). Compared to study participants, those who refused to participate had a significantly different gender distribution (*p* = 0.0309), with a greater proportion of men, but a similar age distribution (*p* > 0.05).

### Participants’ characteristics

More than half of the participants were ostomy patients (55.9%), who had an average age of 65.5 years old (SD = 12.9). Men represented 65.9% of this group. The majority were retired/pensioners (75.5%). The sociodemographic characteristics of the participants with ostomy, including those of dependent ostomy patients, whose information was indirectly obtained from their caregivers, are summarized in the next section.

The caregivers were mostly women (81.7%), who had an average age of 54.8 years old (SD = 14.4). More than half were professionally active (54.5%). Almost one-third were retired/pensioners (31.5%), and one in 10 were unemployed. They were mainly acquiring products for one of their parents/parents-in-law (40.2%) or their spouses (38.5%). Table 1 summarizes the caregivers’ characteristics.

**Table 1:** Sociodemographic characteristics of caregivers

Variables		Caregivers
		*n* = 181
**Sex,** ***n*** **(%)**	Male	32 (18.3%)
	Female	143 (81.7%)
		*n* = 175; NR^a^ = 6
**Age, years**	Average (SD)	54.8 (14.4)
	Median	55
	Minimum-maximum	21–93
		*n* = 173; NR^a^ = 8
**Employment situation,** ***n*** **(%)**	Retired/Pensioner	97 (54.5%)
	Employed	56 (31.5%)
	Unemployed	18 (10.1%)
	Other	7 (3.9%)
		*n* = 181
**Kinship/relationship with the ostomy patient,** ***n*** **(%)**	Daughter/son or daughter/son-in-law	72 (40.2%)
	Spouse/partner	69 (38.5%)
	Nephew/niece	10 (5.6%)
	Healthcare professional	8 (4.5%)
	Brother/sister	6 (3.4%)
	Father/mother or parent-in-law	6 (3.4%)
	Other	8 (4.5%)
		*n* = 179; NR^a^ = 2

^a^ Non-respondents

### Ostomy patients’ sociodemographic characteristics

People living with a stoma were mostly men (61.6%) and had a mean age of 68.1 years (SD = 14.3). Almost 80% were retired/pensioners, while 13.0% were employed and 5.1% were unemployed. Table 2 summarizes the sociodemographic characteristics of all ostomy patients by the type of respondent.

**Table 2:** Sociodemographic characteristics of ostomy patients, according to the type of respondent

Variables		Autonomous ostomy patients	Dependent ostomy patients	All
		*n* = 229 (55.9%)	*n* = 181 (44.1%)	*n* = 412 (100%)
**Sex,** ***n*** **(%)**	Male	151 (65.9%)	80 (54.4%)	233 (61.6%)
	Female	78 (34.1%)	67 (45.6%)	145 (38.4%)
		*n* = 229	*n* = 147; NR^a^ = 34	*n* = 378; NR^a^ = 34
**Age, years**	Average (SD)	65.5 (12.9)	72.2 (15.5)	68.1 (14.3)
	Median	67	76	70
	Minimum-maximum	23–95	8–99	8–99
		*n* = 221; NR^a^ = 8	*n* = 147; NR^a^ = 34	*n* = 368; NR^a^ = 44
**Employment situation,** ***n*** **(%)**	Retired/pensioner	173 (75.5%)	124 (84.4%)	297 (79.0%)
	Employed	42 (18.3%)	7 (4.8%)	49 (13.0%)
	Unemployed	10 (4.4%)	9 (6.1%)	19 (5.1%)
	Other	4 (1.7%)	7 (4.8%)	11 (2.9%)
		*n* = 229	*n =* 147; NR^a^ = 34	*n* = 376; NR^a^ = 36

^a^ Non-respondents

Ostomy patients with a caregiver were significantly older than the autonomous patients (*p* < 0.0001). The autonomous group had a higher proportion of men (*p* = 0.0152) and employed subjects (*p* = 0.0009) than the dependent group (18.4% vs. 4.8%).

### Ostomy patients’ clinical characteristics

Intestinal ostomy (78.3%) and urostomy (19.0%) were the most frequent types of ostomy in our sample (Table 3). Six participants (1.5%) reported having both an intestinal and urinary stoma. Most ostomies were permanent (81.7%). On average, the time since surgery was 5.9 years (SD = 6.6). The most recent intervention had occurred less than 1 month before data collection, and the longest observed time since surgery was 40 years.

**Table 3:** Ostomy patients’ clinical characteristics

Variables		All
		*n* = 412
**Ostomy type,** ***n*** **(%)**^**a**^	Intestinal	322 (78.3%)
	Urinary	78 (19.0%)
	Respiratory	16 (3.9%)
	Other (gastrostomy)	1 (0.2%)
		*n* = 417
**Ostomy cause*, n*** **(%)**^**a**^	Tumour	306 (75.0%)
	Inflammatory disease	33 (8.1%)
	Obstruction	24 (5.9%)
	Trauma	9 (2.2%)
	Organ malformation	7 (1.7%)
	Other	35 (8.6%)
		*n* = 414; NR^b^ = 4
**Expected duration,** ***n*** **(%)**	Temporary	73 (18.3%)
	Definitive	326 (81.7%)
		*n* = 399; NR^b^ = 13
**Time since intervention,** ***n*** **(%)**	Average, years (SD)	6 (6.6)
	Median, years	3
	< 1 year	74 (19.0%)
	1–5 years	176 (45.2%)
	≥ 6 years	139 (35.7%)
		*n* = 389; NR^b^ = 23

^a^ There may be multiple answers per subject

^b^ Non-respondents

The youngest ostomy patient underwent surgery in their first year of life, and the eldest underwent surgery at 96.5 years old. Tumours were the main reason for surgery in every age class. Inflammatory diseases and organ malformations were more common in the younger age groups.

**Fig. 1:**
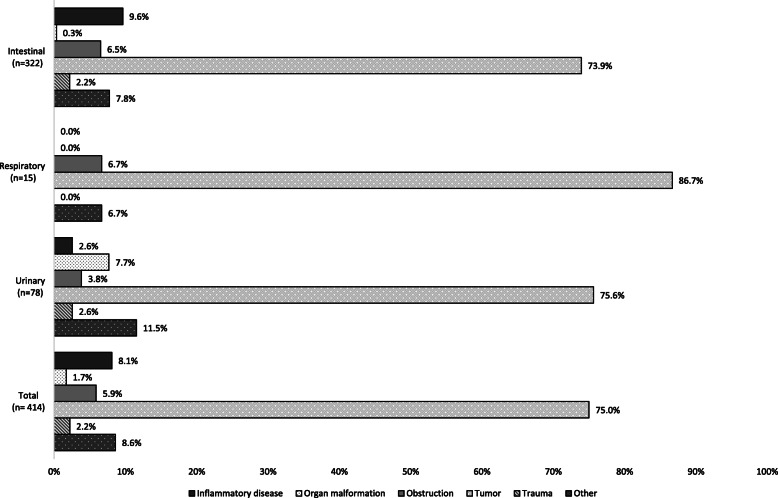
Cause of surgery according to the type of ostomy

Men represented 61.4% of patients with intestinal ostomies, 56.9% of patients with urinary ostomies and 86.7% of patients with respiratory ostomies. Three-quarters (75.0%) of ostomies resulted from oncologic diseases. Intestinal ostomies were also a consequence of inflammatory diseases (9.6%) and obstructions (6.5%), while 7.7% of urinary interventions occurred due to organ malformations. Trauma accounted for 2.2% of all surgeries (Fig. 1).

Approximately half of ostomy patients (48.2%) had access to a specialized nurse in stoma care. No significant differences were found between ostomy patients with and without specialized nurse care regarding the degree of urbanization of the pharmacy setting where they were recruited (*p* > 0.05).

### Access to pharmacies and ostomy products

Most of the participants (97.3%) acquired ostomy products at the pharmacy where they usually purchased other medicines. Approximately 55% of the respondents indicated that they could not obtain all the products they needed in their first visit to the pharmacy due to unavailability. In those cases, more than half (54.9%) said they were able to access the desired products later that same day. Overall, 72.3% of respondents usually had some ostomy products stocked at home.

### Satisfaction with pharmacies

**Fig. 2:**
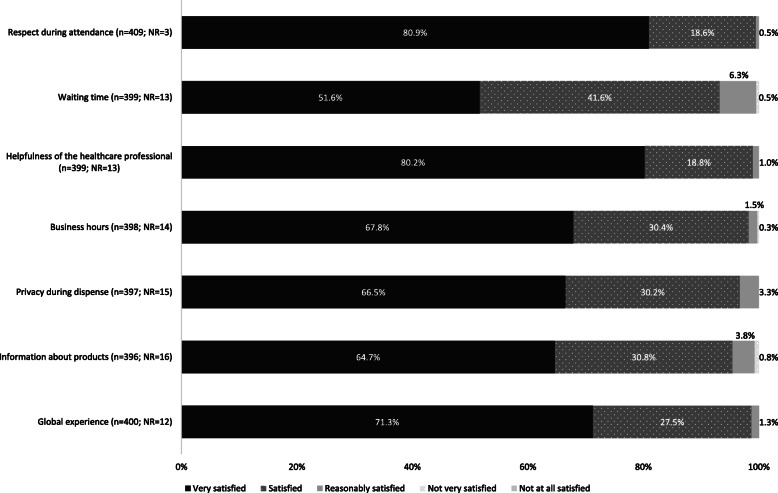
Participants’ satisfaction with pharmacies

In all the evaluated dimensions, more than 93% of the participants were either *satisfied* or *very satisfied* with the dispensing of ostomy products at the pharmacy (Fig. 2). Participants gave the highest scores to ‘respect during attendance’ and ‘helpfulness of the healthcare professional’. The dimension with the lowest score was ‘waiting time’.

### Expectations with pharmacists and pharmacies

Most of the participants agreed that community pharmacists can provide information about stoma care (87.2%) and diet (84.8%). If the participants gave a negative response, they were asked to justify their answers. Of those who did not agree, more than half mentioned that these healthcare professionals lack knowledge on these subjects (stoma care: 59.4%; diet: 63.4%).

**Fig. 3:**
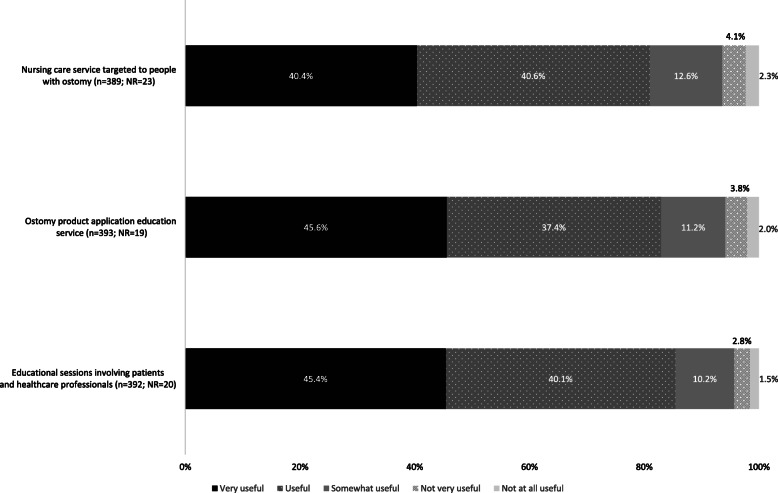
Participants’ evaluation of the utility of potential new ostomy services at pharmacies

Participants were also asked to rate the utility of three potential new ostomy services provided at the pharmacy: ‘stoma care nursing service’, ‘ostomy product application education service’ and ‘educational sessions involving patients and healthcare professionals’. More than 80% of the respondents thought that the ‘ostomy product application education service’ would be either *useful* or *very useful*. ‘Educational sessions involving patients and healthcare professionals’ received the highest score, and ‘stoma care nursing service’ received the lowest based on the proportions of *very useful* or *useful* responses (Fig. 3). No significant differences were found between the responses of ostomy patients and caregivers (*p* > 0.05). In the comparison of the scores based on the expected duration of the stoma, more people with temporary stomas saw little or no utility (*not very useful*, *not useful at all*) in the ‘educational sessions’ service.

## Discussion

This study provides a characterization of people with ostomy and caregivers who usually buy ostomy products at Portuguese community pharmacies. Moreover, it allows the evaluation of this population’s perceptions and expectations with their pharmacists and pharmacies. Approximately half (44.1%) of the study participants were caregivers, which suggests that stomas – or their underlying causes – may limit ostomy patients’ access to pharmacies. Most caregivers were employed women who purchased products for direct family members. A previous study on the profile of informal caregivers of elderly people in Portugal found that caregivers were mostly family members of the patient (mainly wives or daughters/daughters-in-law) aged between 45 and 55 years old (for daughters/daughters-in-law) or 65 years or older (for wives) [13], which is consistent with our findings.

The average age of the ostomy patients was 68.1 years, and most were male (61.6%) and retired/pensioners (79.0%). These characteristics are consistent with the findings of an international study – the Ostomy Life Study [14]. However, there is also literature indicating an even distribution between genders in the ostomy patient population [15, 16]. The higher proportion of men with an ostomy in our study may be explained by the aetiology of the surgery, as discussed below. On average, ostomy patients with caregivers were approximately 7 years older than those in the autonomous group (72.2 vs. 65.5 years). As ageing diminishes individuals’ health and autonomy [17], an increased need for a caregiver/representative is expected in elderly patients.

The prevalences of intestinal and urinary ostomy in our sample were 78.3 and 19.0%, respectively, in accordance with the Ostomy Life Study, which reported similar prevalences for the various ostomy types - ileostomy (37%) and colostomy (43%) (intestinal ostomies) and urostomy (18%) [14].

On average, the time since surgery was 5.9 years (SD = 6.6), and permanent stomas were the most prevalent (81.7%), which is in line with the findings of Andersen [15]. Approximately 75% of the interventions were a consequence of oncologic events, regardless of age and ostomy type. Inflammatory diseases were the second most prevalent reason for surgery, with a much lower prevalence (8.1%). Several studies have indicated the same pathologies as the main causes for stoma surgery, but in different proportions [15, 16]. Since tumours were the main reason for ostomy in our sample, these differences may be explained by epidemiological data on the neoplasms that usually lead to this intervention. The Institute for Health Metrics and Evaluation (IHME) reported that since the early 2000s, the prevalence of colorectal and bladder cancer has been increasing in Portugal. Compared to the OECD countries, Portugal has a higher prevalence of both types of cancer [18]. Differences between the referred regions have been increasing throughout the years and have been more pronounced in males, which may be a plausible reason for the higher proportion of intestinal and bladder ostomies in men in Portugal. Furthermore, the all-cancer incidence rate is higher in men than in women in all OECD countries [19].

Regarding access to ostomy products, half of the participants (54.9%) reported that they could not obtain every product they needed in the same visit to the pharmacy. As the supply system is relatively efficient and wholesalers can deliver the products in a matter of hours, pharmacies usually do not stock every product and instead order them from wholesalers as needed. Considering this result, communication between pharmacies and customers should be improved to avoid unnecessary pharmacy visits.

Regarding satisfaction with pharmacies and their dispensing of ostomy products, more than 93% of the participants were either *satisfied* or *very satisfied* with all the evaluated domains. These results are in agreement with Policarpo et al. [20], who found that the majority of the Portuguese population is satisfied and has a positive relationship with pharmacies.

Respondents also acknowledged pharmacists’ ability to advise about stoma care and diet. Nevertheless, compared to the results of a study conducted at the time of the new dispensing model implementation, our study results on participants’ perceptions of pharmacist advice regarding diet were significantly different [11]. A higher proportion of negative responses was obtained, suggesting that customers may have experienced some gaps in pharmacists’ knowledge in the 2 years that followed the implementation of the new dispensing model. This finding suggests that pharmacists may need more training regarding this subject.

More than 90% of participants thought that pharmacists could provide information about stoma care and skin complications. As such, pharmacists have an opportunity to intervene and potentially reduce expenses associated with ostomy complications and hospitalizations. Notably, a previous study found that approximately 36.3% of ostomy patients developed peristomal skin complications within 90 days after surgery, leading to longer hospital stays, readmissions and increased health expenditures [16]. Furthermore, a systematic review concluded that peristomal skin complications were the most incident type of stoma-related morbidity, regardless of the type of ostomy [21]. Some European countries already offer ostomy-related services. English community pharmacies provide a ‘stoma appliance customization’ service, which is classified as an advanced service in the NHS and is therefore funded on a national level and performed by accredited pharmacists in community pharmacies [22]. In Scotland, pharmacies can provide a ‘stoma service’ that aims to dispense, customize and deliver ostomy products to patients and is funded by the Scottish Government [23]. A similar service could be tested in Portuguese community pharmacies. In the development process, aspects such as users’ expectations, acceptability, and willingness to pay should be considered, and an effectiveness and economic evaluation study should be conducted.

Participants gave higher scores to the proposed ‘educational sessions involving patients and healthcare professionals’, expressing a desire for greater knowledge and tools to take an active role in disease management. Patients with temporary stomas gave the highest proportion of negative scores to this service, possibly because they expected the condition to have a brief and less significant impact on their lives than those with permanent stomas [24]. The Canadian Society of Ostomy Nurses strongly recommends ostomy patients be followed up before and after the procedure [25]. However, in Portugal, only 48.2% of ostomy patients receive care from a specialized nurse. The poor access levels might be due to the scarcity of nurses with this specialization rather than to geography, as there were no significant differences among those with and without specialized nursing care. Pharmacies and nurses could form a partnership to provide this service for those who cannot otherwise access it.

### Limitations

Participating pharmacies were found to be similar in terms of setting and region to the population of pharmacies. This suggests that results may be generalised. However, the study has some limitations and its findings must be interpreted with special care considering the data collection methodology, and inherent biases.

Because pharmacy participation was not mandatory, self-selection bias could have occurred. Moreover, it was assumed that individuals who went to the pharmacy and acquired ostomy products for someone else were caregivers. Ostomy patient characterization and further analysis were conducted based on the assumption that those who went to the pharmacy on their own were “autonomous” and that those with a caregiver were “dependent”. Given that this was a cross-sectional study, this classification may have been biased, as there is a chance that ostomy patients may have gone to the pharmacies by themselves despite having a caregiver, or vice versa.

Clinical data were self-reported, which could involve some degree of inaccuracy, especially for the caregivers; however, it is assumed that patients and caregivers were aware of the type and duration of the ostomy and the reason for surgery.

## Conclusions

This study provides the sociodemographic and clinical characteristics of ostomy patients and their caregivers living in Portugal and contributes to filling the knowledge gap regarding this population. It also provides information regarding the relationship between patients, caregivers, and pharmacies. Due to their proximity to the patients, community pharmacies are in a good position to provide counselling in ostomy management and stoma care. Pharmacists have an opportunity to expand their roles in ostomy management and meet patients’ and caregivers’ needs to establish a closer bond with the community over time.

## Supplementary information

**Additional file 1.** “Ostomy_Questionnaire_EN version” – Translation of the original study questionnaire to English language.

## Data Availability

The data that support the findings of this study are available from the corresponding author upon reasonable request.

## Abbreviations

ANF: National Association of Pharmacies, Portugal
IHME: Institute for Health Metrics and Evaluation
NHS: National Health Service
OECD: Organisation for Economic Co-operation and Development
