# Supplementary material for: Who are the ostomy patients and caregivers attending Portuguese community pharmacies? A cross-sectional study
Source: BMC Health Serv Res. 2020 Oct 2;20:914. doi: 10.1186/s12913-020-05765-7 (PMC7532616; doi:10.1186/s12913-020-05765-7)
Supplement: Supplementary file 1 — Additional file 1. “Ostomy_Questionnaire_EN version” – Translation of the original study questionnaire to English language. [file 12913_2020_5765_MOESM1_ESM.docx]

**CEFAR Ostomy and Community Pharmacies II**

| **To be filled in by the pharmacist:**  Date: ____/____/2019 **Pharmacy ID**: _________ Patient ID: ______ (sequential number, e.g., 01) |
| --- |

**Self-administered questionnaire**

**STUDY OBJECTIVES**

The Centre for Health Evaluation & Research (CEFAR) of the National Association of Pharmacies is conducting a study that aims to briefly characterize people living with a stoma and/or their caregivers who frequently visit the pharmacy, as well as assess their satisfaction with and expectations of their relationship with the pharmacy.

The questionnaire is **self-administered and anonymous**. For each question presented, please mark with an X the option that best suits you. You should choose the answer that best represents your reality and understanding.

**I. PARTICIPANT’S CHARACTERISTICS (ostomy patient and/or caregiver)**

2. Please select the most appropriate option:

I am the person living with a stoma (ostomy patient) 🡪 Fill in Box I

I am the caregiver of someone living with a stoma 🡪 Fill in Boxes I and II

| **BOX I** |  | **BOX II** |
| --- | --- | --- |
| ***Ostomy patient’s*** *characteristics* |  | ***Caregiver’s*** *characteristics* |
| 1. Sex:   Female  Male |  | 1. Sex:   Female  Male |
| 1. Age: _____ years |  | 1. Age: ______ years |
| 1. Employment status:   Student  Employed  Unemployed  Pensioner/Retired  Other, please specify: _____________ |  | 1. Employment status:   Student  Employed  Unemployed  Pensioner/Retired  Other, please specify: _____________ |
| - If you are the representative/caregiver of the ostomy patient, please also fill in BOX II *(and then proceed to the following section)*. - If you are the ostomy patient, please proceed to the following section, *Section* *II. OSTOMY CHARACTERISTICS* |  | 1. What is **your** kinship/relationship with the ostomy patient?   Spouse  Parent/Parent-in-law  Son/daughter  Neighbour  Other, please specify: _____________ |

**II. OSTOMY CHARACTERISTICS**

1. Which type of ostomy are you buying these products for?

Respiratory (tracheostomy)

Intestinal (opening in the digestive tract – ileostomy, colostomy, etc.)

Urinary (opening in the urinary tract – urostomy, etc.)

Other, please specify: ________________________________________________________

1. What led to the creation of the stoma?

Inflammatory disease (e.g., Crohn’s disease, colitis)

Organ malformation

Obstruction

Tumour

Trauma

Other, please specify: ________________________________________________________

1. What is the expected duration of the stoma?

Temporary

Permanent

1. How long ago did the surgery take place? ________months **OR** ________years

**III. ACCESS TO A MEDICAL PRESCRIPTION FOR OSTOMY PRODUCTS**

1. How often do you need to request a prescription for the ostomy products? *(select only 1 option):*

Once to twice a year

Three to four times a year (once every 3 months)

Once every 2 months

Monthly

More than once a month

I don’t know

1. On average, how long does it take to get the prescription from the moment you request it? *(select only 1 option)*

I can access the prescription on the same day I request it

1 to 2 working days after I request it

3 to 5 working days after I request it

Over a week after I request it

**IV. ACCESS TO THE PHARMACY AND OSTOMY PRODUCTS**

1. Is the pharmacy where you buy your ostomy products the same pharmacy where you normally buy your medicines?

Yes

No

1. Are you able to collect all the prescribed products in the same pharmacy visit?

Yes

No

- 1. If you answered ‘**No**’, how long do you have to wait to get all the products? *(select only 1 option)*

A few hours (less than 1 day)

1 to 2 days

3 to 5 days

Over 5 days

1. Since you started acquiring ostomy products at the pharmacy, have you experienced any shortage or unavailability?

Yes

No

- 1. If you answered ‘**Yes**’, can you please specify the products and how much time you had to wait until you accessed them? __________________________________________________

___________________________________________________________________________

1. Do you usually keep a stock of ostomy products at home in case of unavailability?

Yes

No

**V. SATISFACTION WITH THE PHARMACY**

1. How would you rate your satisfaction level with the dispensing of ostomy products in the pharmacy regarding the following aspects? *(select only 1 option per row)*

|  | Not at all satisfied | Not very satisfied | Reasonably satisfied | Satisfied | Very satisfied |
| --- | --- | --- | --- | --- | --- |
| Respect during attendance |  |  |  |  |  |
| Waiting time |  |  |  |  |  |
| Helpfulness of the healthcare professional who dispenses the products |  |  |  |  |  |
| Business hours |  |  |  |  |  |
| Privacy during dispensing |  |  |  |  |  |
| Information about the dispensed products |  |  |  |  |  |
| Overall experience with the attendance and dispensing service |  |  |  |  |  |

**VI. EXPECTATIONS WITH THE PHARMACY**

1. Do you think that pharmacists can help inform you about the stoma and surrounding skin care?

Yes

No

- 1. If you answered ‘**No**’, why?

Because they lack knowledge on ostomy skin care and complication signs.

Because they are never available to address this issue.

Other, please specify: ___________________________________________________________

1. Do you think that pharmacists can provide information and answer any questions about the proper diet for ostomy patients?

Yes

No

- 1. If you answered ‘**No**’, why?

Because they lack knowledge on the proper diet for ostomy patients.

Because they are never available to address this issue.

Other, please specify: _____________________________________________________

1. Are you followed up by a nurse on your stoma care?

No

Yes

- 1. If you answered ‘**Yes**’, would you say that your nurse has specialized knowledge on this subject?

Yes

No

I don’t know

1. How would you rate the following ostomy services in terms of utility, if they were to be provided in community pharmacies? *(select only 1 option per row)*

|  | Not at all useful | Not very useful | Somewhat useful | Useful | Very useful |
| --- | --- | --- | --- | --- | --- |
| Nursing care service targeted at people with ostomy |  |  |  |  |  |
| Education service on ostomy product application |  |  |  |  |  |
| Education and knowledge promotion sessions on ostomy involving patients and health professionals |  |  |  |  |  |

**We kindly ask you to place the folded questionnaire in the envelope provided for that purpose. Close the envelope and return it to the pharmacist.**

**Thank you for participating!**
